# PCR-Based Detection and Genotyping of *Helicobacter pylori* in Endoscopic Biopsy Samples from Brazilian Patients

**DOI:** 10.1155/2013/951034

**Published:** 2013-01-16

**Authors:** Silvia M. Ferreira Menoni, Sandra Helena Alves Bonon, José Murilo Robilota Zeitune, Sandra Cecília Botelho Costa

**Affiliations:** ^1^Department of Internal Medicine, Faculty of Medical Sciences, State University of Campinas (UNICAMP), 13081-970 Campinas, SP, Brazil; ^2^Nursing Course, Federal University of Mato Grosso do Sul (UFMS), 79620-080 Três Lagoas, MS, Brazil; ^3^Gastrocentro, State University of Campinas (UNICAMP), 13083-878 Campinas, SP, Brazil

## Abstract

*Helicobacter pylori* (*H. pylori*) is considered the second most prevalent infection in man. A precise diagnosis is important for treating patients with the indicative gastrointestinal symptoms. The present study analyzes the effectiveness of a molecular biology method (PCR) comparing the results obtained with the histology and with the rapid urease tests. PCR was used in the detection and genotyping of the *H. pylori* urease-C gene and the patterns which were obtained from the patients studied. 141 biopsy samples from 131 patients were evaluated. 59 paraffin biopsies samples were positive for *H. pylori* according to the histological examination. Of those, 59/12 (20.3%) were amplified using PCR. Of the 82 samples from the fresh biopsies, 64 were positive for *H. pylori* according to the rapid urease test (78%); there was an agreement of 100% with PCR. Sixty positive *H. pylori* samples were genotyped (58 samples of fresh biopsies and 2 samples of paraffin biopsies) using two restriction enzymes. The patterns observed were analyzed with the computational program BIO 1D; 11 patterns with the enzyme *HhaI* and 12 patterns with the enzyme *MboI* were found. However, it was not possible to find a statistically significant correlation between the specific genotypes and digestive pathologies. Accordingly, future research should be performed to confirm a statistically significant relationship between genotyping and gastrointestinal symptoms.

## 1. Introduction

The *Helicobacter pylori* (*H*. *pylori*) infection is currently endemic worldwide with high prevalence (up to 60%) in developing regions such as South America. The infection causes chronic Gastritis, gastric and duodenal ulcers, gastric adenocarcinoma, and mucosa-associated lymphoid tissue [1–6]. *H*. *pylori* is associated with several autoimmune diseases, including idiopathic thrombocytopenic purpura (ITP), Sjögren syndrome, systemic sclerosis [7], Graves' disease [8], and autoimmune pancreatitis [9]. As a result of this association with autoimmune diseases, we hypothesized that *H*. *pylori* might induce systemic immunological changes. Although the seroprevalence of *H. pylori* may be high in the normal population, a minority develops peptic ulcers [10, 11]. Some possibilities could justify this data: genetic differences in the host's environmental factors and bacterial strains.

A variety of tests are now available to diagnose *H. pylori* infection. Histological examination of gastric tissue, bacterial cultures, rapid urease test, use of DNA probes, and PCR analysis, when used to test gastric tissue, all require endoscopy. In contrast, breath tests, serology, gastric juice PCR, and urinary excretion of N^15^ ammonia are noninvasive tests that do not require endoscopy. PCR offers high sensitivity and specificity as a technique for the detection of *H. pylori* although the accuracy of such techniques varies widely [12]. The aim of this work is to analyze the effectiveness of the molecular biology method PCR in the detection of *H. pylori* in patients with gastrointestinal symptoms, comparing the results with the histology and the rapid urease test and using the PCR-RFLP technique to detect *H. pylori* subtypes in endoscopic biopsy samples obtained from Brazilian patients.

## 2. Material and Methods 

### 2.1. Patients

141 samples were collected from 131 patients with several diagnoses of gastrointestinal pathologies. Among them, 99 patients who were involved in this study had Gastritis, 29 had ulcers, 5 had Gastritis and ulcers, 3 had esophagitis, and 5 had other gastrointestinal diseases. The patients were 48 years old on average; their ages varied from 4 to 90 years old. 81 were males and 50 females (Table 1). All patients were submitted to an endoscopy at the Gastrocentro (Center of Digestive Tract Studies), University Hospital, State University of Campinas, SP, Brazil, after informed consent was obtained and protocol approved by the Hospital's Ethics Committee.

### 2.2. Methods

The methods used for the detection of *H. pylori* were polymerase chain reaction (PCR) and PCR-RFLP for genotyping. They were chosen in order to detect the bacterium and its subtypes in endoscopic biopsies of fresh tissues and paraffin tissues. The fresh biopsy samples were conserved in physiologic serum 0.9% until the DNA was extracted. At least two, 5 to 10 mm, ribbons of paraffin were collected from the paraffin tissue. In the fresh biopsies, at least two fragments were collected.

#### 2.2.1. DNA Extraction—Gastric Paraffin Biopsy

DNA extraction from the endoscopic biopsies fastened in paraffin followed the method described by Davis et al., 1995 [13], with some modifications. 

At least two, 5 to 10 mm, ribbons of paraffin were placed in a 1.5 mL Eppendorf tube. One mL of xylene was added to the samples. They were shaken, allowed to rest for 3 to 5 minutes, and then centrifuged for 5 minutes, discarding the xylene afterwards. After three washes in 100%, 95%, and 70% ethanol, respectively, the samples were dried at room temperature. Next, the material was resuspended in a solution of Proteinase K, 50 mM Tris, 0.5% SDS, and sterile water. 430 *μ*L of phenol were added to the sample, which was homogenized and centrifuged for another 30 minutes at 14,000 RPM. The supernatant containing DNA was transferred to a new tube and 430 *μ*L of phenol/chloroform (1 : 1) were added and centrifuged again for 5 minutes at 14,000 RPM. Chloroform/isoamyl ethanol was added (24 : 1) to the supernatant, which was homogenized and centrifuged for another 30 minutes at 14,000 RPM. After the addition of 75 *μ*L of ammonium acetate and 750 *μ*L of 100%, ethanol samples were inverted several times and incubated overnight to −20°C. After centrifugation for 30 minutes at 12,000 RPM to −4°C, the supernatant was discarded. The precipitate was carefully washed with 500 *μ*L of chilled 70% ethanol, which was immediately discarded. The material was dried at room temperature and resuspended in a solution containing 50 mL of sterile water, 10 M of Tris (pH 8.0), and 1 mL of EDTA and stored at −20°C until its use.

#### 2.2.2. DNA Extraction: Fresh Biopsy

Firstly, a fresh 3 to 7 mm biopsy section was placed in a 1.5 mL sterile tube with 190 *μ*L of a solution that contained 0.1 M of Tris HCl (pH 7.5) and 1% of SDS. Secondly, 10 *μ*L of proteinase K were added (10 mg/mL) to the solution. The sample was macerated and incubated overnight at 55°C. After that, 200 *μ*L of phenol and 200 *μ*L of both chloroform and isoamyl alcohol (24 : 1) were added. The solution was then homogenized and centrifuged for one minute. Next, the supernatant was removed, and 200 *μ*L of chloroform/isoamyl alcohol were added, homogenized, and centrifuged for 1 minute. Next, the supernatant was removed again, and 25 *μ*L of sodium acetate 3 M and 900 *μ*L of 100% ethanol at −20°C were added; after vortexing the mixture, it was incubated for 30 minutes at −70°C. The samples were centrifuged for 15 minutes at 15,000 RPM. The supernatant was discarded. Lastly, the DNA was resuspended in 25 *μ*L of distilled and sterile water. [14].

#### 2.2.3. PCR Amplification of the *H. pylori*

The polymerase chain reaction followed the method described by Saiki and col. [15], with some modifications. 

For each amplification reaction, 0.5 to 0.7 *μ*L of the DNA under investigation were used, for a total reaction volume of 20.0 *μ*L. The reaction buffer contained 50 mM KCL, 10 mM Tris-HCL pH 8.4, 2.5 mM MgCL_2_, 2.0 pmol of each “primer,” 200 *μ*M of each deoxynucleotide triphosphate (dATP, dCTP, dGTP, and dTTP), 2.5 units of Taq polymerase (Gibco-BRL), and sufficient water to give the total volume of 20.0 *μ*L. The reaction mixture was covered with 100 *μ*L of mineral oil and the tubes were placed in a DNA Thermal Cycler (Perkin-Elmer). 

**Figure 1:**
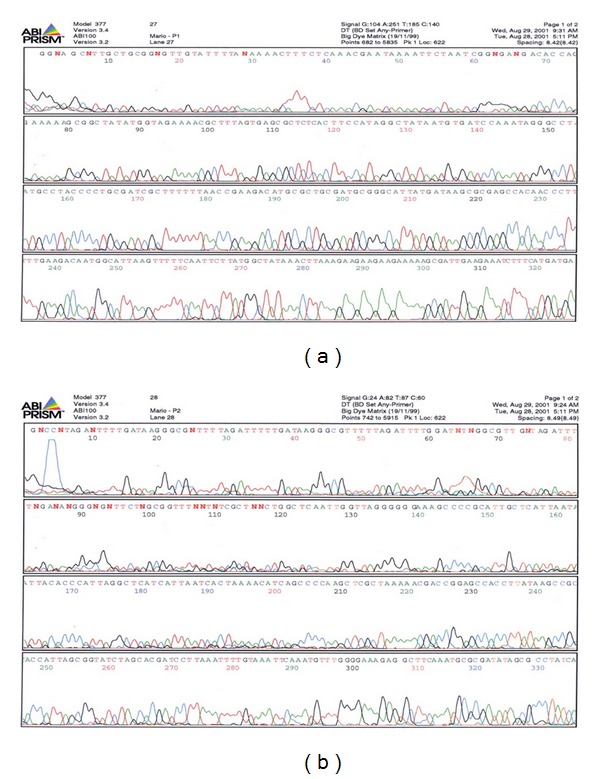
Automated sequencing (Abi Prism 377) for the ureC region of *H. pylori*. Positive samples for *H. pylori* infection obtained from PCR in order to prove that the sequences being amplified did belong to the genetic sequence of a DNA segment of the urease-C area of *H. pylori (enzymes HhaI and MboI)*. All DNA samples taken from the patients presented genetic sequences similar to the one of *H. pylori*, as described for the urease-C area of the Gene Bank (I Square 1).

The reactions that followed were found to be optimal. The samples were heated to 94°C for 60 s to denature the DNA, cooled to 57°C for 90 s to allow the primers and the DNA to reanneal, and then heated to 72°C for 120 s for primer extension. By the final cycle, the extension period was 7 min. A total of 40 cycles were performed. The amplified product was detected by direct gel analysis. 5 *μ*L of the reaction mixture were subjected to electrophoresis with 2% agarose minigel, and the DNA was visualized using UV fluorescence after staining with ethidium bromide. Molecular weight markers were included in each gel. An 820 base-pair band was seen when samples were amplified using primers P1 and P2 to detect *H. pylori* (Table 1 and Figure 1). 

#### 2.2.4. PCR-Based Restriction Fragment Length Polymorphism Typing of *Helicobacter pylori* (RFLP)

After the amplification was confirmed, the PCR product was submitted to digestion with the restriction enzymes HhaI and MboI for fragmentation of Urease-C [16]. 

**Figure 2:**
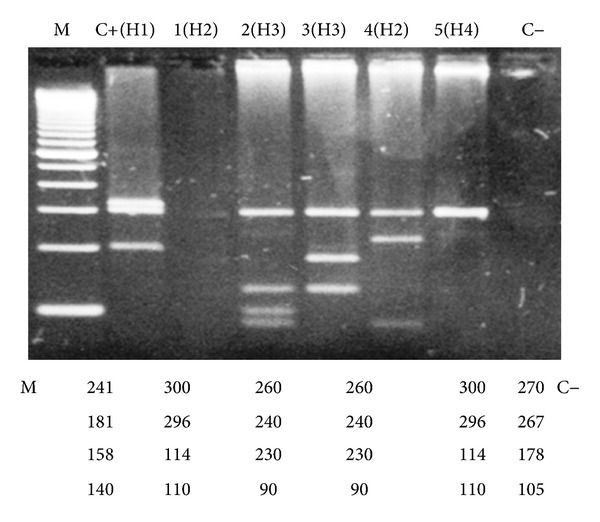
Digestion patterns with the enzyme *HhaI* found in some analyzed samples. Electrophoresis in 2% agarose gel 1000, stained with bromide ethidium. M marker of molecular weight, C+ (1), 1(H2), 2(H3), 3(H4), 4(H4), 5(H2), C-negative control. Note: the most frequent pattern found was H4, with 14 patients (25.8%).

**Figure 3:**
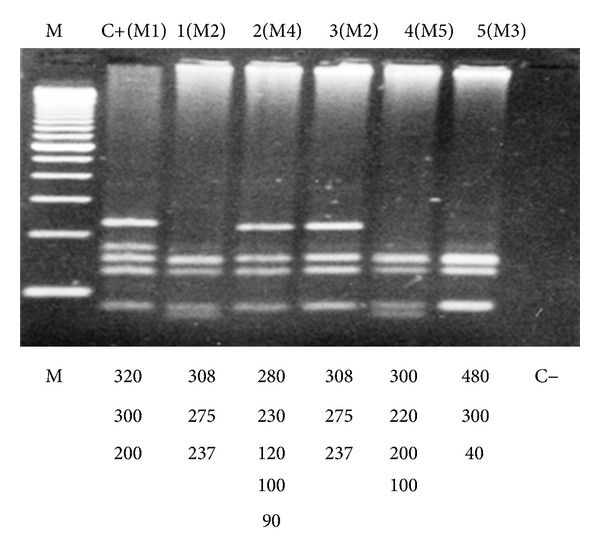
Digestion patterns with the enzyme *MboI* found in some analyzed samples. Electrophoresis in 2% agarose gel 1000, stained with ethidium bromide. M marker of molecular weight; C+(M1); 1(M2); 2(M4); 3(M2); 4(M5); 5(M3); C-negative control. Note 1: Pattern M4 was the most frequent, with 15 patients (25.8%). Note 2: patient 1(M2) presented very clear bands in this analysis, but in a posterior analysis it was possible to classify this patient in group M2.

The fragments which were produced were submitted to electrophoresis in a 2% gel agarose 1000 (Gibco-BRL), stained with ethidium bromide, visualized under ultraviolet light and photographed in Polaroid System. The patterns which were found were compared and analyzed with the computational program Bio 1D (Analysis of Restriction—PCR-RFLP—Restriction Fragment Length Polymorphism), version 99 (Vilber Loumart) (Figures 2 and 3).

Approximately 10 *μ*L of the amplified product were used for the digestion process which also contained 2.0 *μ*L of the corresponding enzyme. Water was added to fill 20.0 *μ*L and the mixture was placed in a 37°C bath overnight. 

#### 2.2.5. Automatic Sequencing

Automatic sequencing was performed using the program Abi Prism, model 377, version 3.4, and Abi 100, version 3.2. Sequencing allowed for the identification of the studied DNA region (Primers P1 and P2). Figure 1 shows the automatic sequencing, proving that the sequence is *Helicobacter pylori.*

## 3. Results 

A total of 141 endoscopic biopsy samples from 131 patients were studied for *H. pylori* infections with PCR and the results were compared with Urease and Histology tests. 82/64 (78%) fresh samples had a positive Urease test for *H. pylori*. A PCR test detected all of the 64 positive samples identified by the Urease test (100%) (Table 2).

Fifty-nine paraffin biopsies, all found to be positive through a histological examination, were submitted to the DNA extraction procedure and Beta-Globin PCR to prove the quality and the presence of DNA in the extracted samples. Only 14/59 (23.7%) samples were positive for the Beta Globin gene, but in two of them *H. pylori* was not amplified by PCR, even though they had a positive Histology test (Table 3). In the other 45 samples, it was impossible to detect Beta Globin in the DNA using PCR, primarily because of the low amount of paraffin samples and/or because the reaction was inhibited due to paraffin and xilol in the extraction procedures. No contamination occurred and the samples were tested two times.

Among the 141 fresh endoscopic biopsy samples, 58 were tested using the RFLP technique to detect the different *H. pylori* strainswith the restriction enzymes *HhaI* and *MboI*. All 58 samples showed positive PCR for Beta Globin and *H. pylori* genes. The product obtained from the *H. pylori* amplification gene by direct PCR was 820 base pairs. Eleven digestion patterns for *HhaI* and twelve for MboI were found (Table 4). The most frequent patterns were HhaI–3 with 12.58 (18.3%), HhaI-4 with 14.58 (23.3%), MboI-2 with 13.58 (21.7%), and MboI-4, with 15.58 (23.3%). The median age was 45, 39, 39, and 57, respectively, in each of the detected patterns. The most frequent diseases in the patients of this study were Gastritis and ulcers.

## 4. Discussion 

Gastric cancer is one of neoplasms that cause the majority of deaths not only in Brazil but all over the world. The type of cancer caused by *H. pylori *could be linked to gastric chronic. Differences in the degree of virulence between strains have lead to an increased risk of developing gastric diseases [17]. 

The *H. pylori* infection is distributed in a cosmopolitan way, reaching mainly the adult population of low socioeconomic levels in developing countries. The discharge infection rate is correlated with bacterial virulence and inherent factors of the particular host, mainly with respect to the immune system [18]. 

 It should be noticed that the route of fecal-oral transmission appears to be the biggest problem in the prevalence of infection, making *H*.* pylori* a serious public health problem in both developed and developing countries [19].

The present study analyzes the effectiveness of the molecular biology method PCR in the detection of *H. pylori* in patients with gastrointestinal symptoms, comparing it with the histology and rapid urease test. 

The polymerase chain reaction (PCR) for the diagnosis of *H. pylori* is a very sensitive and specific method [20], providing fast and safe diagnosis. Many results indicate that PCR sensitivity is close to that of culture tests [21], but for verifying the eradication of *H. pylori* the effectiveness of PCR can be markedly superior [22, 23]. The methodology used in other studies to distinguish the different *H. pylori* subtypes has been PCR-RFLP [24] that through analyzis of the PCR product with restriction endonucleases that resulting fragments of different sizes and the digestion profile is decisive to define the strains. The restriction enzymes HhaI and MboI were used for the Urease-C area [16]. The extreme degree of variability observed among the strains of *H. pylori* became an important focus of scientific attention, as the investigators recognized the significant impact that this phenomenon can have on several research areas, such as the development of vaccines, the development of resistance to antimicrobial agents, and the study of the pathogen-host interaction [25, 26]. Considering that 10 to 20% of people infected with *H. pylori* develop obvious diseases, the reliable identification of the lineages could actually be very beneficial [27]. Previous studies that have used several techniques characterized *H. pylori* as a highly variable species that presents countless lineages, each one with its own and different genotype [28–31]. 

The genotyping of *H. pylori* is important for characterizing the most pathogenic genotype and the most frequent strain. This information can be used for clinical and epidemic studies. Even if many infections are clinically silent, the organism infected with *H. pylori* presents increased morbidity and mortality [5, 32, 33].

In the present study we standardized PCR with material obtained from the fresh endoscopic biopsies samples of patients attending Gastrocentro (the Center of Digestive Tract Studies), Medical School, UNICAMP. Some of the gastric biopsy samples were collected in paraffin and some were not. With regard to the standardization of the DNA extraction technique from the paraffin biopsy samples, several difficulties were found, because the samples contained a small amount of tissue fragments and many of the paraffin samples did not amplify the *β*-Globin gene, demonstrating degraded DNA of poor or inhibited quality.

PCR was used because it is more specific and faster when compared to other methods; the product of PCR can be processed with restriction enzymes to verify *H. Pylori *strains. Besides, starting with the PCR, DNA sequencing can be made to verify mutations, which no other technique is capable of doing.

As an internal control of the reaction was used in all samples (human *β*-Globin gene), in the fresh-air biopsy samples positive for *H. pylori*, we had 100% PCR amplification. However, in several paraffin samples, the *β*-Globin did not amplify, indicating an inefficient DNA extraction of the samples. 

The efficiency of *H. pylori* detection PCR in fresh samples was superior to that in the paraffin samples. We suspect that PCR inhibition may have occurred due to the method used in DNA extraction from paraffin or the fact that that the samples were insufficient.

The extraction of DNA from fresh samples had excellent results. Among the 82 analyzed samples, 64 were positive and 18 negative, with 100% in agreement with PCR. 

In the present study, we used the PCR-RFLP method for the differentiation of *H. pylori* strains from specimens obtained from gastric biopsies taken from Brazilian patients. Using this methodology we observed that 12 and 11 patterns were produced, respectively, by the two restriction enzymes *MboI* and *HhaI* from 58 specimens obtained from gastric biopsies. Two were samples of biopsies in paraffin and 58 were samples of nonfastened gastric biopsies (Table 1). 

This data suggests that genotyping using PCR-RFLP can be useful as a fast procedure for the specific identification of *H. pylori* lineages in gastric biopsies specimens [16]. Several protocols of genotyping analysis were proposed for distinguishing the lineages of clinically isolated *H. pylori* [34–38]. Several primer pairs were described for detection and the typing of *H. pylori* was based on the amplification of the ureA [34], ureA plus ureB [35], and ureC genes [36, 38]. These results demonstrate great diversity in the urease genes in clinical *H. pylori *samples. Li et al. [16] found 3, 11, and 6 different patterns which were produced by 19 clinically isolated samples, respectively, digested by the restriction enzymes *HhaI, MboI* and AluI. Foxall et al. [35] found 10 different patterns which were produced by 22 clinically isolated samples, when the restriction enzyme *HaeIII* digested the PCR product of 2.4 Kb which had been amplified by the ureA and ureB genes. Lopez et al. [37] found that the patterns generated by the digestion of PCR products with the *HaeIII* enzyme, starting from ureA and ureB, were almost as different as the standard *HaeIII*. Akopyanz et al. [28] found 18 *MboI *and 27 *HaeIII* RFLP patterns, PCR products amplified by ureA and ureB genes of 2.4 Kb of 60 *H. pylori* lineages, and that the patterns distinguished 44 separate groups. Each isolated group did not differ from the other ones in the RFLP analyses of ureA and ureB products, but differed in *MboI* digestion of the 1.7 Kb ureC and ureD segments. Such a fact indicates that PCR-RFLP analyses of ureC genes can produce a great number of standard RFLPs.

Several studies have confirmed that PCR-RFLP analysis of the ureC gene can differentiate clinically isolated *H. pylori*. Using restriction endonucleases, Moore et al. [38] analyzed the 1.1 Kb portion of the ureC gene amplified by the “PCR” of 21 clinically isolated *H. pylori*. The samples were divided into four groups after digestion with the enzyme *HindIII*, while the lineages were divided into 15 groups after they were digested with the enzymes *AluI* and *PvuI.* Fujimoto et al. [36] demonstrated that the digestion of 820 bp of the *H. pylori* ureC gene with the restriction enzymes *HhaI*, *MboI,* and *MseI* resulted in 10, 10, and 11 different patterns, respectively. Dooley et al. [3] used three types of enzymes in the PCR product of a 1.179 bp portion of the *H. pylori* ureC gene. Eleven, 10, and 6 digestion patterns were produced by the *HhaI, MboI,* and *AluI* enzymes, respectively.

In our study we used two types of restriction enzymes in the amplified products of 820 pb of the *H. pylori* ureC gene. We obtained 11 and 12 different patterns, respectively, from the 58 clinically isolated samples which were studied. Our results suggest that the PCR-RFLP analysis of this portion of the *H. pylori* ureC gene is a reliable method, and that genotyping of PCR in this area can be used for epidemic studies and for the differentiation of isolated *H. pylori strands*.

 This study also revealed a high level of genetic diversity isolated in the different *H. pylori* positive patients studied in Brazil.

The obtained genotyping patterns were compared using the computational program Bio 1D. We found 11 patterns with the *HhaI* enzyme and 12 patterns with the *MboI* enzyme. The reason for the small number of studied samples was due to the fact that it was not possible to establish a significant statistical correlation between specific digestive pathologies and standard genotypes.

We believe that the genotyping of *H. pylori* can contribute to the study of the microorganism's characteristics, facilitating the detection of pathogenic or nonpathogenic strains and, in turn, providing a better understanding of the several virulence factors that the bacterium uses to cause diseases. 

### 4.1. Statistics

Percentage agreement was calculated to compare *H. pylori* genotypes obtained from PCR performed directly on gastric biopsies, with the genotypes obtained from the PCR of DNA extracted from paraffin and fresh samples, as well as histology and urease tests.

## Figures and Tables

**Table 1:** Patient characteristics.

	Total (*n* = 141)
Patients	
Sex (male/female)	81/50
Age years (median)	48 years (range 4–90)
Disease	
Gastritis	99 (70.2%)
Ulcers	29 (20.6%)
Gastritis + ulcers	5 (3.5%)
Esophagitis	3 (2%)
Other*	5 (3.5%)

*Inflammation, duodenitis, and splenomegaly.

**Table 2:** Comparison between PCR and urease test in fresh biopsy samples.

	Urease test	PCR
Positive	64	64
Negative	18	18
Total	82	82

100% agreement.

**Table 3:** Comparison between PCR and histology test in paraffin biopsy samples.

	Histology	PCR
Positive	59	12
Negative	0	47

Total	59	59

Positive *Beta Globin* (DNA detection)	—	14
Negative *Beta Globin* (no DNA detection)	—	45

Total	0	59

*Two paraffin samples were positive for the *Beta Globin* gene, but negative for the *H. pylori* gene.

**Table 4:** Use of the Restriction Fragment Length Products (RFLP) technique for genotyping the positive *H. pylori* PCR products using restriction enzymes (*HhaI* and *MboI*).

Restriction enzyme	Frequency (%)	Disease	Median age (years)
HhaI-1	1/58 ( 1.7)	Gastritis + ulcers	41
HhaI-2	5/58 (8.6)	3 Gastritis; 2 ulcers	29
**HhaI**-**3**	**12/58 (20.7)**	**9 Gastritis**;** 3 ulcers**;	**45**
**HhaI**-**4**	**14/58 (24.1)**	**8 Gastritis**;** 3 ulcers**;** 1 esophagitis**;** 1 Inflamation**	**39**
HhaI-5	3/58 (5.2)	1 Gastritis; 2 ulcers	54
HhaI-6	6/58 (10.3)	3 Gastritis; 2 ulcers; 1 Gastritis + ulcers	39
HhaI-7	1/58 (1.7)	1 ulcers	19
HhaI-8	4/58 (6.9)	1 Gastritis; 2 ulcers; 1 esophagitis	48
HhaI-9	2/58 (3.4)	1 Gastritis; 1 ulcers	37
HhaI-10	2/58 (3.4)	2 ulcers	72
HhaI-11	8/58 (13.8)	6 Gastritis; 1 inflamation; 1 ulcers	54
MboI-1	2/58 (3.4)	2 Gastritis	72
**MboI**-**2**	**13/58 (22.4)**	**7 Gastritis**;** 5 ulcers**,** 1 esophagitis**	**39**
MboI-3	8/58 (13.8)	3 Gastritis; 3 ulcers; 1 inflamation	49
**MboI**-**4**	**15/58 (25.8)**	**10 Gastritis**;** 5 ulcers**	**57**
MboI-5	4/58 (6.9)	1 Gastritis; 1 ulcers; 1 esophagitis; 1 Gastritis ulcers	27
MboI-6	2/58 (3.4)	1 Gastritis; 1 ulcers	37
MboI-7	4/58 (6.9)	1 Gastritis, 1 splenomegaly; 2 ulcers	50
MboI-8	1/58 (1.7)	1 Gastritis	45
MboI-9	5/58 (8.6)	2 Gastritis; 2 ulcers; 1 Gastritis + ulcers	47
MboI-10	2/58 (3.4)	2 Gastritis	28
MboI-11	1/58 (1.7)	1 Gastritis	31
MboI-12	1/58 (1.7)	1 Gastritis	38
